# Low‐Salt Diet Induces Claudin‐3 Expression and Drives Adaptive Changes in Collecting Duct of Claudin‐3‐Deficient Mice

**DOI:** 10.1111/apha.70216

**Published:** 2026-04-03

**Authors:** Ali Sassi, Alexandra Chassot, Sara Jellali, Nicolas Liaudet, Ana Polat, Felix Baier, Deborah Stroka, Mikio Furuse, Eric Feraille

**Affiliations:** ^1^ Department of Cellular Physiology and Metabolism, Faculty of Medicine University of Geneva Geneva Switzerland; ^2^ Bioimaging Core Facility, Faculty of Medicine University of Geneva Geneva Switzerland; ^3^ Visceral Surgery and Medicine, Inselspital, Bern University Hospital, Department for BioMedical Research University of Bern Bern Switzerland; ^4^ Division of Cell Structure National Institute for Physiological Sciences Okazaki Japan

**Keywords:** aldosterone, claudin‐3, collecting duct, kidney, low‐salt diet, sodium transport

## Abstract

**Aim:**

Renal sodium reabsorption occurs through both transcellular and paracellular pathways. Tight junction proteins play a key role in mediating paracellular transport. The collecting duct is critical for the fine tuning of sodium balance and is highly responsive to changes in dietary salt intake. This study aimed to determine whether a low‐sodium diet modulates paracellular sodium permeability by regulating the expression or localization of claudin‐3, a major tight junction protein in the collecting duct.

**Methods:**

Wild‐type and claudin‐3 knockout male mice were fed low (0.01%) or normal (0.18%) sodium diets for 7 days, with or without treatment with spironolactone, a mineralocorticoid receptor antagonist. The expression of tight junction proteins was analyzed by immunoblotting and immunofluorescence. Functional effects of claudin‐3 on ion permeability were evaluated in cultured mouse collecting duct principal cells using chamber recordings after claudin‐3 overexpression or gene silencing.

**Results:**

Low‐sodium diet increases claudin‐3 expression in mouse kidneys. In cultured cells, aldosterone enhanced claudin‐3 abundance and its plasma membrane localization. Claudin‐3 overexpression reduced, while its silencing increased paracellular permeability to sodium and chloride. Claudin‐3 knockout mice on a low‐sodium diet compensated by upregulating epithelial sodium channel subunits, claudin‐4, claudin‐8, and claudin‐10. This adaptive response persisted under mineralocorticoid receptor blockade.

**Conclusions:**

Our findings demonstrate that aldosterone strengthens the paracellular sodium barrier in the collecting duct by inducing claudin‐3. In the absence of claudin‐3, compensatory regulation of other claudins and sodium transporters preserves sodium homeostasis under low‐salt conditions, thus revealing adaptive mechanisms in renal sodium handling.

## Summary

- Aldosterone regulates sodium transport in the collecting duct not only through epithelial sodium channels but also by strengthening the paracellular barrier via claudin‐3 upregulation, thereby reducing paracellular sodium‐chloride back‐leak.
- The compensatory responses observed in claudin‐3‐deficient mice reveal a coordinated interaction between transcellular and paracellular mechanisms that together maintain sodium homeostasis under low‐salt conditions.
- These findings highlight the dynamic and hormone‐sensitive nature of the tight junction complex in renal epithelia, offering new perspectives for understanding and managing disorders of sodium balance.

## 1 Introduction

The regulation of sodium (Na^+^) transport by the kidney is essential for maintaining electrolyte balance, fluid homeostasis, and blood pressure. The collecting duct (CD), the final site of regulated Na^+^ reabsorption along the kidney tubule, plays a crucial role in this process. Sodium transport in the CD occurs primarily through the epithelial sodium channel (ENaC) located at the apical membrane and the Na,K‐ATPase located at the basolateral membrane, the latter generating the electrochemical gradient necessary for transcellular Na^+^ reabsorption [1, 2]. In addition to transcellular transport, paracellular transport is highly dependent on tight junctions (TJ), which may generate either a specific Na + permeability or a barrier that prevents passive Na^+^ back‐leak into the tubular lumen and thereby maintain a net Na^+^ reabsorption process [3]. TJ are composed of various proteins, including occludin, junctional adhesion molecules (JAMs), and claudins. Claudins are the primary determinants of TJ selectivity, regulating the permeability of ions and water across epithelial barriers [4, 5]. The expression profile of claudins in the kidney is highly segment‐specific, with distinct claudins contributing to either barrier function or selective ion permeability [6, 7].

Aldosterone, the main mineralocorticoid hormone, is a key regulator of Na^+^ balance and renal sodium reabsorption. In CD principal cells, binding of aldosterone to the mineralocorticoid receptor (MR) triggers genomic and non‐genomic responses that enhance Na^+^ transport [8, 9, 10]. Aldosterone increases the expression and activity of both ENaC and Na,K‐ATPase, thereby enhancing transcellular Na^+^ reabsorption [11, 12, 13, 14]. However, aldosterone‐induced Na^+^ reabsorption generates a lumen‐negative transepithelial potential, together with the Na^+^ gradient between the interstitial space and the lumen, which may drive a Na^+^ back‐leak through the paracellular pathway. Increased paracellular Na^+^ back‐leak potentially compromises the efficiency of Na^+^ reabsorption. Aldosterone has been reported to strengthen the paracellular barrier by modulating TJ composition. For example, it increases claudin‐8 expression in the kidney and colon, reinforcing the Na^+^ diffusion barrier. However, the extent to which aldosterone regulates the amounts of other claudin species along the kidney tubule TJ remains to be fully established. Claudin‐3 is widely expressed in epithelial tissues, including the respiratory, urinary, and gastrointestinal tracts, as well as the liver, salivary, and mammary glands. Its function remains controversial: some studies suggest that claudin‐3 enhances barrier function by reducing paracellular permeability, while others suggest that it may facilitate selective permeability pathways depending on the tissue and physiological context [15, 16, 17, 18, 19, 20, 21, 22]. The function of claudin‐3 has been well studied in a few key tissues (e.g., liver and brain), while its specific role in the kidney remains poorly understood and requires further investigation. In this study, we investigated the effect of aldosterone on claudin‐3 expression in CD principal cells. Using in vivo and in vitro models, we demonstrated that aldosterone upregulates claudin‐3 protein abundance, suggesting a potential role for claudin‐3 in the hormonal regulation of paracellular Na^+^ transport. Furthermore, we examined the functional consequences of claudin‐3 loss by studying claudin‐3‐deficient mice under a low‐salt diet, which stimulates endogenous aldosterone secretion. These mice exhibited compensatory adaptations, including increased expression of α‐ and γ‐ENaC subunits and upregulation of claudin‐4, claudin‐8, and claudin‐10, indicating that the absence of claudin‐3 triggers specific molecular adjustments to preserve Na^+^ balance under low‐salt diet conditions. These findings provide new insights into the mechanisms regulating renal Na^+^ reabsorption and highlight the importance of TJ regulation in maintaining electrolyte balance.

## 2 Methods

### 2.1 Animals

For the experiments investigating the effects of low‐NaCl diet on claudin‐3 in wild‐type mouse kidneys, male C57BL/6 wild‐type mice (Charles River, Saint‐Germain‐de‐l'Arbresle, France) were used. The generation of the C57BL/6 claudin‐3‐deficient mouse strain with a global claudin‐3 knockout was previously described by Castro Dias et al. [23]. Homozygous claudin‐3 knockout and claudin‐3 wild‐type littermates were obtained by interbreeding heterozygous parents. The mice had ad libitum access to food and water and were maintained on a 12‐h light/dark cycle. All procedures were performed during the light phase in male mice aged 8 to 12 weeks.

To assess the effect of sodium intake, mice were fed for 7 days with either a low‐sodium (Na^+^) diet (0.01% [wt/wt]; LSD) or a normal‐sodium (Na^+^) diet (0.18% [wt/wt]; NSD) (Provimi‐Kliba, Kaiseraugst, Switzerland). One group of mice on the low‐Na^+^ diet received 0.35 mg/100 g body weight/day of spironolactone mixed with food for 7 days.

For physiological parameter measurement, mice were acclimatized to Tecniplast metabolic cages for 24 h. Following acclimation, food intake, water consumption, and urine output were measured over a subsequent 24‐h period.

Venous sinus blood was collected from anesthetized animals using heparinized capillary tubes to prevent clotting. Immediately after collection, the blood samples were analyzed with the Epoc Blood Analysis System (Siemens Healthineers) according to the manufacturer's protocol. This system enabled rapid and reliable measurements of key blood biological parameters.

All animal experiments were approved by the Institutional ethical committee of animal care of the University of Geneva and the cantonal authorities, in accordance with the office of laboratory animal welfare's guidelines for good animal practice. The study also adhered to the standards set by the National Centre for the Replacement, Refinement, and Reduction of Animals in Research (NC3Rs).

### 2.2 Plasma Aldosterone Measurement by Enzyme‐Linked Immunosorbent Assay (ELISA)

Aldosterone concentration in plasma was measured using a commercial competitive ELISA kit (Abnova, KA1883) according to the manufacturer's instructions. Briefly, 100 μL of standards, controls, and plasma samples from wild‐type and claudin‐3 knockout mice were added to wells coated with aldosterone antibody together with an HRP‐conjugated aldosterone antigen. Low‐salt diet samples, which exhibit higher plasma aldosterone levels, were diluted 1:10 in PBS to keep the measurements within the dynamic range of the assay. After incubation at room temperature, wells were washed and tetramethylbenzidine (TMB) substrate was added for color development, followed by addition of stop solution. Absorbance was read at 450 nm, with a reference reading at 625 nm for background subtraction, using a Hidex Sens multimode plate reader, and aldosterone concentrations were calculated by interpolation from a standard curve generated with the supplied standards and internal quality controls.

### 2.3 Cell Culture, Constructs, Viral Particle Production, and Cell Transduction

As previously described, mCCD_cl1_ cells were grown on permeable filters (Transwell, Corning Costar, Cambridge, MA, USA) to confluence in a 1:1 mixture of Dulbecco's modified Eagle's medium and F12 medium, as previously described [24]. Aldosterone (10^−6^ M) was added to the apical and basal compartments 24 h before cell lysis. For gene overexpression, cells were transduced with lentiviruses carrying either an empty vector or wild‐type green fluorescent protein (GFP) as controls, or a construct encoding wild‐type mouse claudin‐3. Both claudin‐3 and GFP were previously subcloned into a modified puromycin‐resistant pSF‐lenti vector (Sigma). For gene silencing, mouse claudin‐3, or scramble short hairpin RNA (shRNA) (Table 1) was inserted into the plasmid pLKO.1 (cat. no 8453; Addgene). pLKO.1 or pSF‐lenti was transiently transfected in packaging HEK293T cells using the Polyplus‐transfection jetPRIME Kit according to the manufacturer's instructions. Lentiviral particles were collected after 72 h and mCCDcl1 cells were transduced. Stable polyclonal cell lines were selected using puromycin (2 μg/mL) which was applied 72 h after transduction.

**TABLE 1:** Sequences of scramble shRNA and shRNAs targeting claudin‐3.

Name	Forward	Reverse
Scramble sh	CCGGGCGCGATAGCGCTAATAATTTCTCGAGAAATTATTAGCGCTATCGCGCTTTTTG	AATTCAAAAAGCGCGATAGCGCTAATAATTTCTCGAGAAATTATTAGCGCTATCGCGC
Cl‐3 sh‐1	CCGGCCAAGCCGAATGGACAAAGAACTCGAGTTCTTTGTCCATTCGGCTTGGTTTTTG	AATTCAAAAACCAAGCCGAATGGACAAAGAACTCGAGTTCTTTGTCCATTCGGCTTGG
Cl‐3 sh‐2	CCGGCCAACACCATCATCAGGGATTCTCGAGAATCCCTGATGATGGTGTTGGTTTTTG	AATTCAAAAACCAACACCATCATCAGGGATTCTCGAGAATCCCTGATGATGGTGTTGG

### 2.4 Electrophysiological Assessment of Paracellular Permeability in mCCD_cl1_ Cell Monolayers

To determine paracellular permeability, measurements were performed using cultured mCCD_cl1_ cells plated onto Snapwell polyester filters (cat. no. 3801, Corning Costar, Cambridge, MA, USA) at a density of 250 000 cells/cm^2^ and maintained in culture medium for 7 days. Following incubation, the Snapwell inserts were detached and positioned in Ussing chambers (model P2300; Physiologic Instruments, San Diego, CA, USA). These chambers were connected to a VCC MC6 multichannel voltage/current clamp (Physiologic Instruments) using silver/silver chloride (Ag/AgCl) electrodes and 3‐M KCl agar bridges.

The apical and basolateral compartments of the Ussing chambers were separately filled with buffer A (120 mM NaCl, 10 mM NaHCO_3_, 5 mM KCl, 1.2 mM CaCl_2_, 1 mM MgCl_2_, and 10 mM Hepes, pH 7.4), with each side containing a total volume of 5 mL. Transepithelial potential differences between the two compartments were recorded using a Quick Data Acquisition DI100 USB board (Physiologic Instruments), while the transepithelial current was maintained at 0 mA throughout the experiment. Prior to measurement, the cells were equilibrated in buffer A for 1 h.

To determine the dilution potential for NaCl, half of the buffer in the basal chamber was replaced with buffer B (240 mM mannitol, 10 mM NaHCO_3_, 5 mM KCl, 1.2 mM CaCl_2_, 1 mM MgCl_2_, and 10 mM Hepes, pH 7.4), ensuring osmolarity balance with mannitol and adjusting pH to 7.4 with HCl. To confirm that ion flux occurred via the paracellular route, 100 μM amiloride (Sigma) and 100 μM 4,4′‐diisothiocyanatostilbene‐2,2′‐disulfonic acid (DIDS; Sigma‐Aldrich) were added to the apical chamber 30 min before measurement. Throughout the experiment, buffers were maintained at 37°C and continuously bubbled with a gas mixture of 95% oxygen and 5% carbon dioxide.

Peak dilution potential values were recorded and used to determine permeability ratios. Absolute ion permeabilities were calculated as previously described [25, 26]. Specifically, absolute permeabilities for Na^+^ and Cl^−^ were computed using the Kimizuka–Koketsu equation, incorporating both the calculated P_Na_/P_Cl_ and the transepithelial resistance measured during the experiment.

### 2.5 Western Blotting

Equal amounts of protein from cultured cells or kidney cortex were separated on SurePAGE precast Bis‐Tris 4%–20% gradient gel (cat. no. M00657, GenScript, Switzerland), and transferred to polyvinylidene difluoride membranes (Immobilion‐P, Millipore, Bedford, MA, USA), as previously described [24]. After incubation with primary antibodies (Table 2), membranes were incubated with anti‐rabbit or anti‐mouse IgG antibody coupled to horseradish peroxidase (Transduction Laboratories, Lexington, KY, USA), the antigen–antibody complexes were detected by enhanced chemiluminescence (Advansta, Menlo Park, CA, USA). Protein abundance was quantified with the image J software. Results are expressed as the ratio of the densitometry of the band of interest to the loading control.

**TABLE 2:** Antibodies used for western blots (WB) and immunofluorescence (IF).

Name	Species	Dilution for WB	Dilution for IF	Supplier	Cat. Number
Claudin‐2	Mouse	1/500		Thermofisher	32‐5600
Claudin‐3	Rabbit	1/500	1/50	Abcam	ab15102
Claudin‐4	Rabbit	1/500	1/100	Cusabio	CSB‐PA275606
Claudin‐7	Rabbit	1/500		Thermofisher	34‐9100
Claudin‐8	Rabbit	1/500	1/100	Thermofisher	40‐0700Z
Claudin‐10	Rabbit	1/500	1/200	Thermofisher	38‐8400
a‐ENaC	Rabbit	1/1000		Prof. J. Loffing	[27]
b‐ENaC	Rabbit	1/500		StressMarq	SPC‐404
g‐ENaC	Rabbit	1/500	1/200	StressMarq	SPC‐405
NKCC2	Rabbit	1/500		Prof. J. Loffing	[27]
NCC	Rabbit	1/500	1/200	Pr J. Loffing	[27]
Aquaporin‐2	Mouse		1/3000	Santa Cruz	sc‐515 770
NHE3	Mouse	1/500		StressMarq	SPC‐400
Megalin	Goat		1/2000	Prof. P. Galichon	[28, 29]
Uromodulin	Rabbit		1/3000	Abcam	Ab207170
Pendrin	Rabbit	1/1000		Prof. C Wagner	[30]
E‐cadherin	Mouse	1/2000		BD Biosciences	610 404
β‐Actin	Mouse	1/10000		Sigma	A5441

### 2.6 Immunofluorescence

Cultured mCCD_cl1_ grown on polycarbonate filters were fixed with ice‐cold methanol for 5 min at −20°C and then washed with PBS during 30 min. Blocking of nonspecific binding sites was done with 10% Normal Goat Serum (NGS) diluted in Phosphate Buffered Saline (PBS). Cells were then incubated overnight at 4°C with antibodies against claudin‐3 diluted 1:100 in 2% BSA followed by a 1 h incubation with Alexa Fluor 488‐conjugated goat anti‐rabbit (cat. no. A‐11008; Invitrogen) diluted 1:1000 and were finally mounted on microscope slides using Vectashield mounting medium (Maravai Life Science, San Diego, CA, USA) with DAPI for nuclear counterstaining. Fluorescence images were acquired using a LSM 700 confocal laser‐scanning microscope (Carl Zeiss, Oberkochen, Germany) using 488‐nm ray lasers. The distance between the Z‐slices was 0.25 μm. From 5 to 10 Z‐stack images were processed per sample using the ZEISS ZEN Imaging Software. ZEN 2.3 (Carl Zeiss, Oberkochen, Germany).

After dehydration and paraffin‐embedding, kidney sections were cut at a thickness of 5 μm. Antigen retrieval was done with Tris‐EDTA buffer 10 mM pH 9. Permeabilization with Triton X100 0.2% in PBS followed by blocking of non‐specific binding sites with 10% NGS diluted in Tris‐buffered saline with 0.1% Tween 20 (TBST) was applied for 1 h. The tissue sections were then incubated overnight at 4°C with primary antibodies (Table 2), in NGS‐TBST followed by a 1 h incubation with a secondary Alexa Fluor 488‐conjugated goat anti‐rabbit (cat. no. A‐11017; Invitrogen) or Cyanin3‐conjugated goat anti‐mouse (cat. no. M30010; Invitrogen) diluted between 1:200 and 1:800 in NGS‐TBST at 37°C. Samples were mounted on microscope slides using Vectashield mounting medium (Maravai Life Science, San Diego, CA, USA). Fluorescence images were acquired using a Zeiss Axio Imager M2 (Carl Zeiss, Oberkochen, Germany). Negative controls were performed in the absence of primary antibody (not shown).

### 2.7 RNA Extraction and Quantitative PCR Analysis

Total RNA was isolated from cultured cells using the EZNA Total RNA Kit I (cat. no. R6834, Omega BIO‐TEK), following the protocol provided by the manufacturer. The RNA concentration and purity were assessed using a Nanodrop spectrophotometer. To generate complementary DNA (cDNA), one microgram of RNA was converted to cDNA using the qScript cDNA SuperMix (Quanta Biosciences), adhering to the manufacturer's guidelines. The specific primers used are detailed in Table 3.

**TABLE 3:** Sequences of primers used for real‐time PCR.

Name	Forward	Reverse
P0	5′‐AATCTCCAGAGGCACCATTG‐3′	5′‐GTTCAGCATGTTCAGCAGTG‐3′
Claudin‐3	5′‐ACATCATCACGTCGCAGAAC‐3′	5′‐TACACCTTGCACTGCATCTG‐3′

For quantitative PCR (qPCR), cDNA was combined with 0.5 μM of each primer and SYBR Green Master Mix (cat. no. A25743, Applied Biosystems, Foster City, CA, USA) and reactions were performed in duplicate using the ABI StepOne sequence detection system (Applied Biosystems). Data analysis was conducted with ABI Prism software (Applied Biosystems), utilizing P0 as an internal reference. The fold change in cDNA levels (*F*) was determined using the equation *F* = 2^(Ct1−Ct2)^, where Ct1 and Ct2 represent the cycle thresholds for the experimental and control conditions, respectively.

### 2.8 Statistics

Results are presented as the mean ± SD from *n* independent experiments. Prism version 10.6.1 (GraphPad Software, San Diego, CA, USA) was used for statistical analysis. The normality of the data was assessed using the Shapiro–Wilk test.

For normally distributed data, statistical differences between two groups were determined using a two‐tailed unpaired Student's *t*‐test and between more than two groups by one‐way ANOVA (followed by appropriate post hoc tests such as Dunnett's, Tukey's, or Sidak's when applicable), whereas for non‐normally distributed data, the non‐parametric Mann–Whitney *U*‐test was used for two groups or the Kruskal–Wallis test for more than two groups. A *p* < 0.05 was considered significant.

## 3 Results

### 3.1 Segment‐Specific Expression of Claudin‐3 Along the Mouse Nephron

To investigate the expression and localization of claudin‐3 in the mouse kidney, we first validated the specificity of the claudin‐3 antibody. This was achieved by Western blot analysis and immunostaining of kidney tissues obtained from both wild‐type and claudin‐3 knockout mice (Figure S1), confirming the antibody's specificity. Immunofluorescence further demonstrated that claudin‐3 is localized as expected at the apical pole of renal epithelial cells and displays the typical reticulated pattern of a tight junctional protein.

**FIGURE 1:**
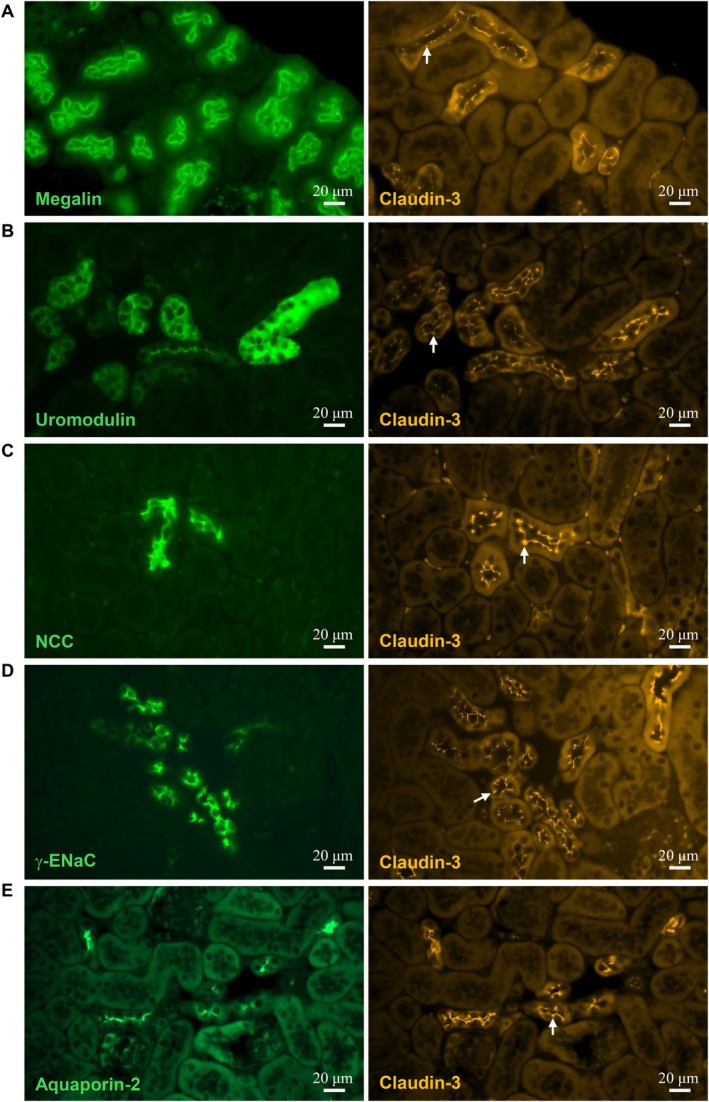
Localization of claudin‐3 along the kidney tubule. Representative immunofluorescence staining of serial kidney sections (×40 objective). A section (*n*) was stained for a specific tubule segment marker (left, green), and the adjacent section (*n* + 1) was stained for claudin‐3 (right, orange, arrows). Segment‐specific markers are megalin for the proximal tubule (A), uromodulin for the thick ascending limb (B), the thiazide‐sensitive sodium‐chloride cotransporter (NCC) for the distal convoluted tubule (C), the epithelial sodium channel gamma subunit (γ‐ENaC) for the connecting tubule and cortical collecting duct (D), and aquaporin‐2 (AQP2) for the collecting duct (E).

As an initial step toward elucidating the physiological function of claudin‐3 in renal epithelia, we sought to determine its segment‐specific distribution along the nephron. Accurate localization of claudin‐3 is critical for understanding its role in the structural and functional organization of the kidney. For this purpose, we performed immunofluorescence labeling on paraffin‐embedded kidney sections from adult C57BL/6 mice, using a panel of well‐characterized segment‐specific markers to identify distinct nephron regions. Claudin‐3 immunoreactivity was not detected in the glomerulus. In the proximal tubule, identified by apical megalin staining, claudin‐3 was likewise absent, indicating that it is not expressed in this segment (Figure 1A). In contrast, claudin‐3 expression was clearly detected in the thick ascending limb (TAL) of Henle's loop, as demonstrated by its colocalization with the TAL‐specific marker uromodulin (Figure 1B). Claudin‐3 was also expressed in the distal convoluted tubule (DCT), on the basis of its colocalization with the sodium‐chloride cotransporter (NCC), a specific marker for this segment (Figure 1C). In the downstream segments of the nephron, including the connecting tubule (CNT) and collecting duct (CD), claudin‐3 was found to colocalize with the γ‐subunit of the epithelial sodium channel (γ‐ENaC), which is expressed in both segments (Figure 1D). To specifically label the CD, we used aquaporin‐2 (AQP2), a marker specific for the collecting duct principal cells. Colocalization of claudin‐3 with AQP2 confirmed its expression along the collecting duct (Figure 1E).

Taken together, these findings indicate that claudin‐3 exhibits a distinct segmental distribution pattern along the nephron. It is selectively expressed in the distal portion of the kidney tubule including TAL, DCT, CNT, and CD, but is absent from both the glomerulus and proximal tubule. This segment‐specific expression suggests that claudin‐3 may play a functional role in epithelial barrier properties and transepithelial transport in the distal nephron.

### 3.2 Aldosterone Upregulates Claudin‐3 in the Mouse Kidney

**FIGURE 2:**
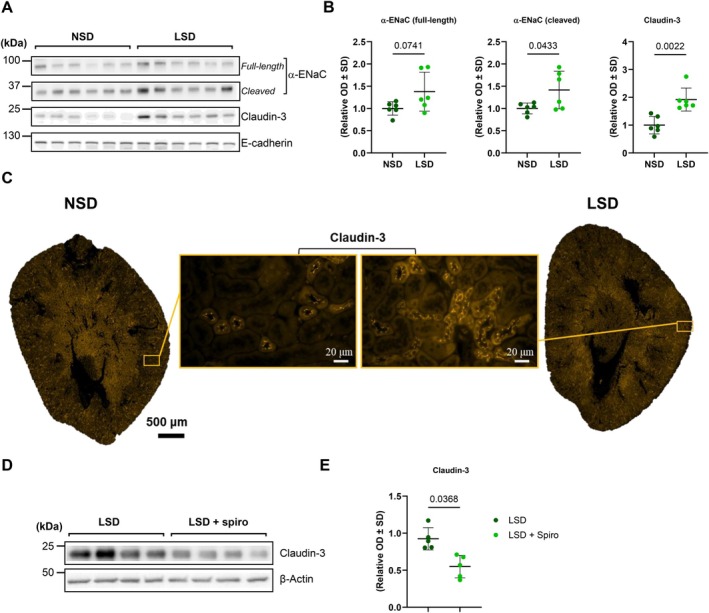
Aldosterone regulates claudin‐3 expression in the mouse kidney. C57BL/6 wild‐type mice were maintained on either a normal‐sodium (Na^+^) diet (0.18%; NSD) or a low‐sodium diet (0.01%; LSD) for 7 days. (A) Western blot analysis illustrating the impact of dietary sodium intake on the abundance of claudin‐3 and α‐epithelial sodium channel (α‐ENaC) in the kidney cortex. Representative blots from six animals per experimental group are shown. (B) Relative densitometric quantification of immunoblots from the kidney cortex, with E‐cadherin used as a loading control. (C) Representative immunofluorescence imaging of claudin‐3 in kidney transverse sections from NSD and LSD mice (×20 objective), shown in orange. Insets of each image are shown with a ×40 objective. (D) Effect of mineralocorticoid receptor blockade on claudin‐3 expression. Mice were fed an LSD (0.01%) and treated or not with spironolactone (0.35 mg/100 g body weight/day) for 1 week before kidney cortex protein extraction. (D) Western blot analysis showing the effect of spironolactone on claudin‐3 expression in the kidney cortex of four animals per experimental group. (E) Densitometric quantification of immunoblots from the kidney cortex of six animals, with β‐actin used as a loading control. Statistical analysis was performed using Mann–Whitney *U*‐test. Data are presented as mean ± SD. kDa: kilodaltons; OD: optical density.

To investigate the potential physiological effect of aldosterone on claudin‐3 in vivo, we analyzed its expression levels in C57BL/6 wild‐type mice subjected to a normal sodium diet (NSD) compared to those fed with a low sodium diet (LSD). Following 7 days of LSD, the expression levels of α‐ENaC were assessed in the renal cortex by Western blot analysis. Both the full‐length and cleaved forms of α‐ENaC were examined. While the cleaved form was significantly upregulated, the full‐length form displayed a trend toward increased expression (Figure 2A,B). These results confirm that LSD leads to an increase in endogenous aldosterone biological activity. Interestingly, claudin‐3 protein abundance, assessed by both Western blot and immunofluorescence, was more abundant in the kidney of LSD‐fed mice (Figure 2A–C). Notably, our previous study demonstrated that LSD induces claudin‐8 expression in mice while it does not affect other collecting duct claudins, including claudin‐4 and claudin‐7 [31].

To further confirm the role of aldosterone in the regulation of claudin‐3 expression, LSD‐fed mice were treated for 7 days with spironolactone, a mineralocorticoid receptor antagonist. The blockade of MR with spironolactone effectively inhibited the LSD‐induced increase in claudin‐3 protein abundance, demonstrating that this effect is dependent on activation of the MR by endogenous aldosterone (Figure 2D,E).

To confirm the effect of aldosterone in vitro, we used mCCD_cl1_ cells, a model of cortical collecting duct principal cells [32]. Treatment with aldosterone (10^−6^ M, 24 h) significantly increased claudin‐3 expression at both protein and mRNA levels, as shown by Western blot, immunofluorescence, and qPCR analyses (Figure S2A–D). Supporting this conclusion, in silico analysis using the Eukaryotic Promoter Database (https://epd.epfl.ch) identified multiple MR‐binding sites in the promoter region of the mouse claudin‐3 gene (Figure S2E), consistent with the genomic effect of aldosterone mediated by the MR. Notably, our previous findings indicated that aldosterone increases claudin‐8 expression in the collecting duct but does not alter the expression of claudin‐4 and claudin‐7, two other claudins expressed in this segment [31].

Functionally, overexpression of claudin‐3 in mCCD_cl1_ cells reduced paracellular permeability to sodium and chloride (Figure S3A–D), while silencing claudin‐3 increased permeability to both ions (Figure S4A–D). These results further demonstrate that claudin‐3 is induced by aldosterone and indicate that it functions as a paracellular sodium‐chloride barrier in CD principal cells.

Together, these findings show that fluctuations in endogenous aldosterone levels regulate claudin‐3 abundance in the mouse kidney and identify claudin‐3 as a functional target of aldosterone in the collecting duct epithelium.

### 3.3 Claudin‐3 Deletion Does Not Alter Basic Physiological Parameters Under Basal Conditions

**FIGURE 3:**
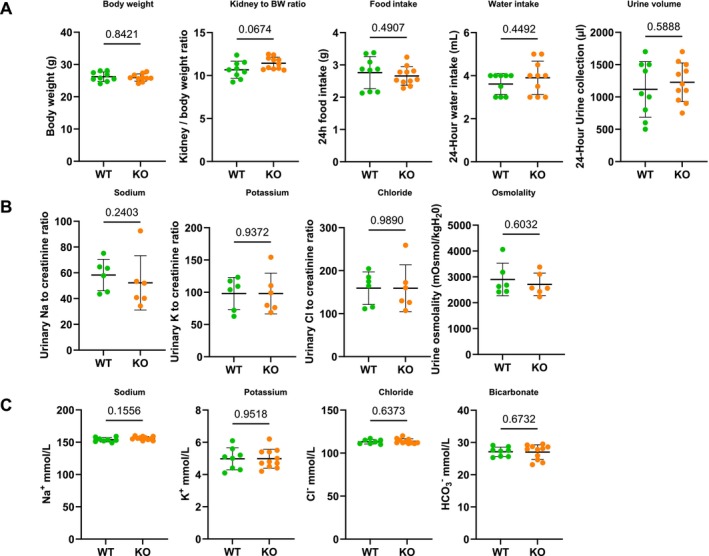
Physiological parameters of claudin‐3‐deficient mice under a normal sodium diet. Mice were maintained on a normal‐sodium diet (0.18%; NSD) and analyzed for various physiological parameters. (A) Mice were first acclimated to the metabolic cages for 24‐h, followed by 24‐h of measurement of physiological parameters, including body weight, kidney to body weight ratio, 24‐h food intake, 24‐h water intake and 24‐h urine volume in WT and claudin‐3 KO mice. (B) Urine was collected over a 24‐h period, and urinary Na^+^, Cl^−^, and K^+^ excretion, as well as urine osmolality, were measured in WT and claudin‐3‐deficient mice. (C) Biological analysis from venous blood showing sodium (Na^+^), potassium (K^+^), chloride (Cl^−^) and bicarbonate (HCO_3_^−^) in WT and claudin‐3 KO mice under NSD. Statistical analysis was performed using the Mann–Whitney *U*‐test. Data are presented as mean ± SD.

To understand claudin‐3 contribution to renal physiology, we analyzed the global claudin‐3 knockout mouse model (KO) [23]. Phenotypic analysis revealed that claudin‐3 deficient mice did not exhibit remarkable phenotypes compared to wild‐type (WT) controls. They remain fertile and maintain normal body weight (Figure 3A). Similarly, the kidney‐to‐body weight ratio at 12 weeks of age did not differ significantly between genotypes (Figure 3A). Basic physiological analysis in metabolic cages did not reveal significant differences in food and water intake or in 24‐h urine output (Figure 3A). Urinary excretion of sodium, potassium, chloride, and basal urine osmolality over 24 h also remained comparable between KO and WT mice (Figure 3B).

Measurements of venous blood gas parameters and plasma electrolyte levels including sodium (Na^+^), potassium (K^+^), chloride (Cl^−^), and bicarbonate (HCO_3_^−^) did not reveal significant differences between the two groups (Figure 3C). These findings indicate that claudin‐3 deficiency does not alter overall growth, metabolic parameters, or body fluid homeostasis under baseline conditions.

### 3.4 Claudin‐3 Knockout Does Not Alter Basal Sodium Transporters or Claudin Expression Along the Nephron

**FIGURE 4:**
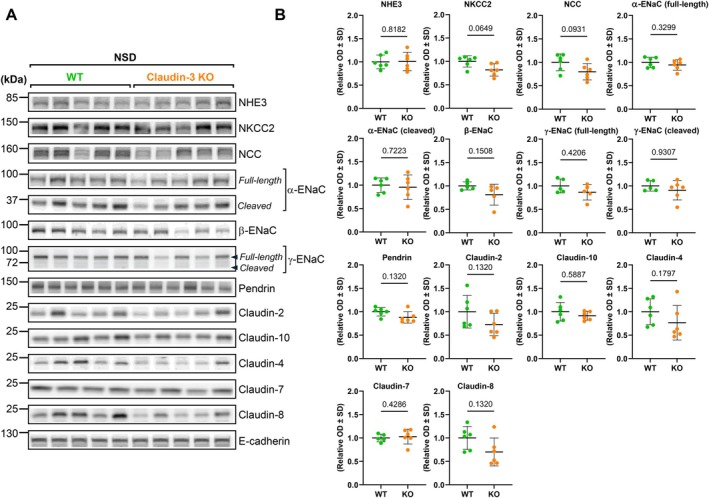
Segmental analysis of sodium transporters and claudins along the nephron in claudin‐3 knockout mice under normal sodium intake. Wild‐type (WT) and claudin‐3 knockout (Claudin‐3 KO) mice were maintained on a normal‐sodium diet (0.18% Na^+^; NSD). (A) Representative Western blots showing the expression of the Na^+^/H^+^ exchanger 3 (NHE3), the Na^+^–K^+^–2Cl^−^ cotransporter (NKCC2), the thiazide‐sensitive sodium‐chloride cotransporter (NCC), full‐length and cleaved forms of the α‐ and γ‐subunits of the epithelial sodium channel (ENaC), β‐ENaC, pendrin, and tight junction claudins (claudin‐2, claudin‐3, claudin‐4, claudin‐7, claudin‐8, and claudin‐10) in the renal cortex of wild‐type and claudin‐3 knockout mice. (B) Densitometric quantification of protein abundance normalized to E‐cadherin from at least five animals per group. Statistical analysis was performed using the Mann–Whitney *U*‐test. Data are presented as mean ± SD. kDa: kilodaltons; OD: optical density.

We then investigated the impact of claudin‐3 deletion on sodium transport and tight junction proteins along the nephron by measuring the expression of key sodium transporters and claudins in proximal and distal segments using Western blot analysis. We assessed the abundance of NHE3, NKCC2, and NCC as well as claudin‐2 and claudin‐10. We found no significant difference between claudin‐3 knockout (KO) and wild‐type (WT) mice for any of these proteins (Figure 4A,B).

The expression of collecting duct‐associated claudins (claudin–4, –7, and –8), epithelial sodium channel (ENaC) subunits (α, β, and γ), and the chloride/bicarbonate exchanger pendrin also remained unchanged. For α‐ENaC and γ‐ENaC, both the full‐length and active cleaved forms were assessed, and no difference between genotypes was observed (Figure 4A,B).

Together, these results demonstrate that claudin‐3 deletion does not induce compensatory changes in claudin expression or sodium transport‐related proteins along the nephron under basal physiological conditions.

### 3.5 Low Salt Diet Induces Segment‐Specific Compensatory Upregulation of ENaC Subunits and Claudins in Claudin‐3‐Deficient Mice

**FIGURE 5:**
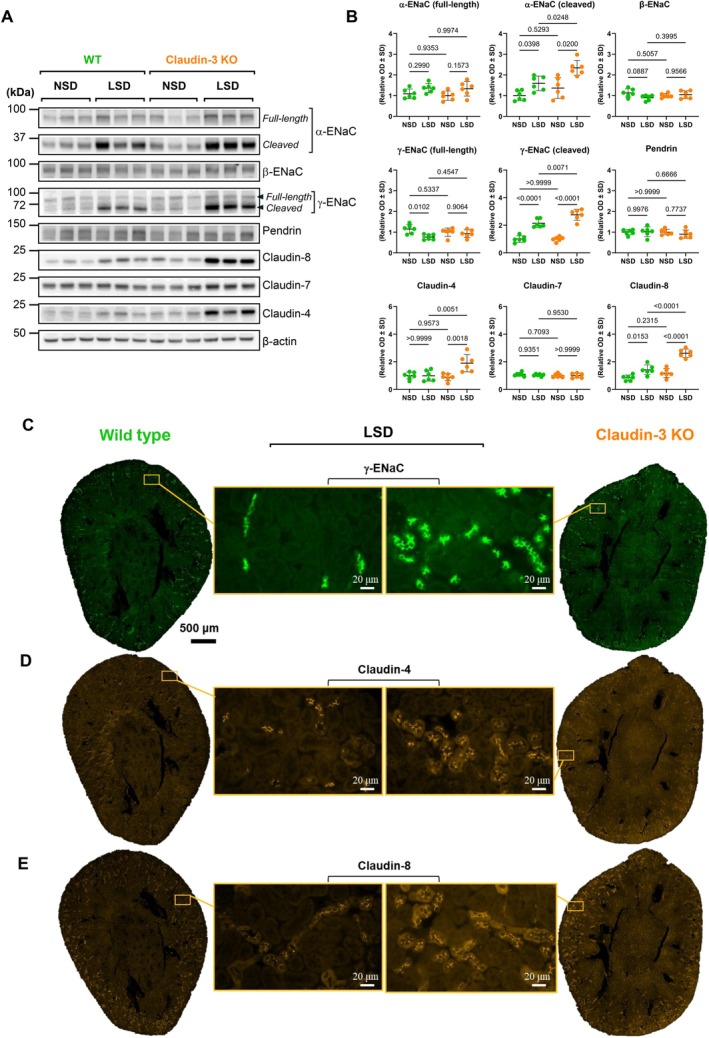
Compensatory upregulation of ENaC subunits and collecting duct claudins in claudin‐3 deficient mice under a low sodium diet. Wild‐type (WT) and claudin‐3 knockout (Claudin‐3 KO) mice were maintained under a normal‐sodium diet (0.18%; NSD) or a low‐sodium diet (0.01%; LSD) for 7 days. (A) Western blot analysis showing the effect of dietary sodium intake on the abundance of α‐epithelial sodium channel (α‐ENaC), γ‐epithelial sodium channel (γ‐ENaC), pendrin, claudin‐4, claudin‐7, and claudin‐8 in the kidney cortex. (B) Relative densitometric quantification of immunoblots from the kidney cortex of six animals, with β‐Actin used as a loading control. (C–E) Representative immunofluorescence images of γ‐ENaC (C), claudin‐4 (D), and claudin‐8 (E) in control and claudin‐3 KO mice (×20 objective) under LSD. Insets of each image are shown with a ×40 objective. Statistical analysis was performed using one‐way ANOVA. Data are presented as mean ± SD. kDa: kilodaltons; OD: optical density.

Since we observed an induction of claudin‐3 expression by aldosterone, we investigated the impact of claudin‐3 deletion on sodium transport under sodium‐restricted conditions to reveal its functional effect in vivo. Claudin‐3 knockout (KO) and wild‐type (WT) mice were fed a low‐salt diet (LSD) for 7 days. LSD induced a significant increase in both cleaved and full‐length forms of α‐ENaC and γ‐ENaC protein levels in the renal cortex of KO compared to WT mice, as shown by Western blot analysis (Figure 5A,B). The upregulation of γ‐ENaC was also confirmed by immunofluorescence (Figure 5C). In contrast, β‐ENaC and Pendrin levels remained unchanged between WT and KO mice (Figure 5A,B).

We also assessed the expression of claudins expressed in the collecting duct, given their role in tight junction integrity and potential involvement in compensatory epithelial responses. Claudin‐4 and claudin‐8 protein abundance was increased under LSD in claudin‐3 KO mice, as shown by Western blot (Figure 5A,B) and immunofluorescence (Figure 5D,E). In contrast, claudin‐7 levels remained unchanged (Figure 5A,B), indicating a selective regulation of claudin expression in response to sodium restriction combined with claudin‐3 deficiency.

**FIGURE 6:**
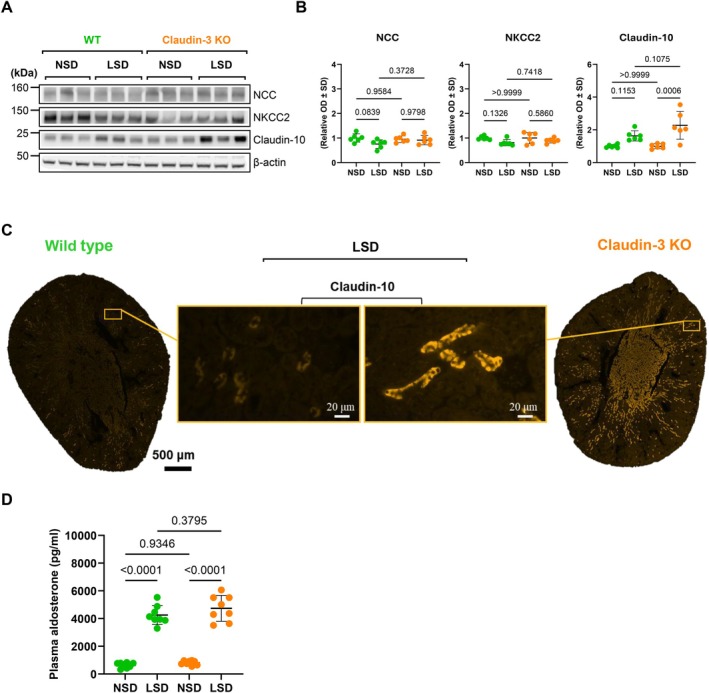
Differential regulation of sodium transporters and claudin‐10 in claudin‐3 deficient mice under a low sodium diet. Wild‐type (WT) and claudin‐3 knockout (Claudin‐3 KO) mice were maintained under a normal‐sodium diet (0.18%; NSD) or a low‐sodium diet (0.01%; LSD) for 7 days. (A) Western blot analysis showing the effect of sodium restriction on NCC, NKCC2, and claudin‐10 in kidney cortex lysates. (B) Densitometric quantification of protein abundance of immunoblots from the kidney cortex of six animals, normalized to β‐actin. (C) Representative immunofluorescence images of claudin‐10 in WT and claudin‐3 KO mice under LSD (shown in orange, ×20 objective). Insets show higher magnification (×40 objective). (D) Plasma aldosterone levels measured by ELISA in WT and claudin‐3 KO mice under NSD and LSD conditions. Statistical comparisons were performed using one‐way ANOVA. kDa, kilodaltons; OD, optical density.

Beyond the collecting duct, we examined other nephron segments where claudin‐3 is also expressed. We analyzed NCC, NKCC2, and claudin‐10 protein abundance by Western blot (Figure 6A,B). No significant difference in NCC levels between WT and KO mice was observed. NKCC2 exhibited a trend toward increased expression in KO mice, but the difference did not reach statistical significance. Importantly, Western‐blot analysis showed that claudin‐10 expression was significantly increased in KO mice. Immunofluorescence analysis confirms this upregulation of claudin‐10 and reveals that it took place along the thick ascending limb of Henle's loop (Figure 6A–C).

To further confirm aldosterone induction under LSD and to determine whether the observed changes could be explained by a stronger endogenous aldosterone response in KO mice, we measured plasma aldosterone levels (Figure 6D). LSD treatment increased plasma aldosterone concentrations as expected, but plasma aldosterone levels did not differ between WT and KO mice.

Together, these results demonstrate that in the absence of claudin‐3, a low‐salt diet triggers segment‐specific compensatory mechanisms, including the upregulation of ENaC subunits in association with a specific set of claudins along the collecting duct and upstream distal nephron segments, likely contributing to the maintenance of sodium balance and epithelial barrier function.

### 3.6 Mechanisms of Adaptation to Low‐Sodium Challenge in Claudin‐3‐Deficient Mice

**FIGURE 7:**
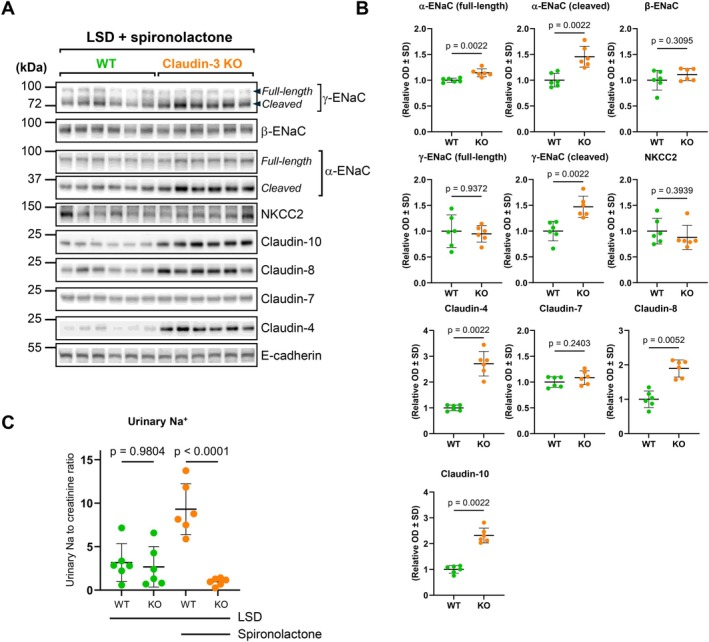
Effect of spironolactone in WT and claudin‐3 deficient mice. Wild‐type (WT) and claudin‐3 knockout (Claudin‐3 KO) mice were fed a low‐sodium diet (0.01%, LSD) and treated with spironolactone (0.35 mg/100 g body weight/day) for 7 days before kidney cortex protein extraction. (A) Representative Western blots showing the abundance of ENaC subunits (α, β, and γ), NKCC2, claudin‐4, claudin‐7, claudin‐8, and claudin‐10 protein abundance in the kidney cortex of six animals per experimental group. (B) Densitometric quantification of immunoblots from (A), normalized to E‐cadherin as a loading control. Data are presented as mean ± SD. (C) Urinary Na^+^ excretion over a 24‐h period measured in WT and claudin‐3 KO mice. Statistical analysis was performed using Mann–Whitney *U*‐test (B) or one‐way ANOVA (C); a *p* < 0.05 was considered significant. kDa, kilodaltons; OD, optical density.

To investigate whether aldosterone‐mineralocorticoid receptor (MR) signaling mediates the compensatory response to a low‐sodium diet (LSD) in claudin‐3 knockout (KO) mice, we treated WT and KO animals with spironolactone, a selective MR antagonist, under LSD conditions. Western blot analysis shows that MR blockade did not abolish the upregulation of both cleaved and full‐length α‐ENaC in KO mice relative to WT (Figure 7A,B). In contrast, the increase in full‐length γ‐ENaC no longer reaches statistical significance under MR antagonism, although cleaved γ‐ENaC remains elevated (Figure 7A,B). In addition, claudin‐4, claudin‐8, and claudin‐10 remained significantly induced in KO mice under MR antagonism (Figure 7A,B). These results suggest that aldosterone does not mediate the adaptations of transcellular and paracellular sodium transport that take place under low sodium diet in claudin‐3 deficient mice.

Urinary sodium measurements (Figure 7C) show that WT mice treated with spironolactone excreted significantly more sodium compared to untreated controls, confirming the efficacy of MR blockade in inhibiting sodium reabsorption. In contrast, KO mice treated with spironolactone did not exhibit increased sodium excretion, supporting the Western blot findings that compensatory sodium transport regulation in KO mice persists despite MR antagonism.

These findings demonstrate that the compensatory upregulation of ENaC subunits and multiple claudins in claudin‐3‐deficient mice on a low‐sodium diet is not exclusively mediated by MR activation. The persistence of α‐ENaC, cleaved γ‐ENaC, claudin‐4, claudin‐8, and claudin‐10 induction under MR blockade strongly suggests an aldosterone‐independent compensatory mechanism aimed at preserving sodium balance.

## 4 Discussion

Claudin‐3, a tight junction protein, is critically involved in renal function through its role in paracellular permeability. Here, we aimed to characterize its in vivo distribution along the nephron, determine its functional significance in sodium handling, and assess its potential regulation by aldosterone.

Our segment‐specific mapping of claudin‐3 along the mouse nephron consolidates and extends previous observations on claudin distribution. Distinct claudin isoforms confer unique paracellular properties to different nephron segments: claudin‐3 is enriched in the distal convoluted tubule, connecting tubule, and collecting duct alongside claudins‐4, –7, and –8 [33]. It is also expressed in the thick ascending limb of Henle's loop where it contributes to barrier tightening [34, 35]. It should be mentioned that in this later segment, claudin‐3 expression is restricted to junctions containing claudin‐16 and claudin‐19 [36]. By definitively placing claudin‐3 at tight junctions in the TAL, DCT, CNT, and CD, and demonstrating its absence from proximal regions, our findings provide the anatomical basis for future functional studies on how claudin‐3 contributes to segment‐specific barrier properties and electrolyte handling in the distal nephron.

Aldosterone is well established as the principal hormonal driver of Na^+^ reabsorption and K^+^ secretion in the aldosterone‐sensitive distal nephron (ASDN), which encompasses the late distal convoluted tubule, connecting tubule and cortical collecting duct [37, 38]. In this context, our findings position claudin‐3 as a novel aldosterone target whose upregulation reinforces paracellular barrier function in the collecting duct. In vitro, aldosterone treatment of mCCD_cl1_ cells increases claudin‐3 abundance and enhances its lateral membrane localization. Claudin‐3 upregulation is associated with a decrease in Na^+^ and Cl^−^ permeability, consistent with observations in MDCK cells reported by Fromm's group [15]. Extending these findings to an in vivo context, we demonstrate that a low sodium diet which is established to increase endogenous aldosterone levels [39, 40] induces a significant increase in claudin‐3 abundance within the renal cortex of mice. This induction is abolished by administration of spironolactone, a mineralocorticoid receptor antagonist, indicating that claudin‐3 upregulation in response to low‐sodium diet is MR‐dependent. Importantly, in a previous study [31], we showed that aldosterone similarly upregulates claudin‐8 in collecting duct epithelia. Collectively, these results support a model wherein aldosterone, acting through MR‐mediated transcriptional mechanisms, enhances tight junction barrier function via selective induction of claudin‐3 and claudin‐8, thereby minimizing paracellular back‐flux of reabsorbed ions. This paracellular reinforcement likely complements canonical stimulation of ENaC to maximize net sodium conservation under conditions of salt depletion and underscores the importance of tight junction remodeling as an underappreciated yet critical mechanism of mineralocorticoid‐driven electrolyte homeostasis.

Our experiments in claudin‐3 knockout mice demonstrate that under baseline conditions, these animals display no detectable alterations in renal function or electrolyte balance, nor changes in sodium transport‐related proteins, including other claudins and ENaC subunits, along the nephron. This lack of phenotype under baseline conditions complements liver studies demonstrating its role as a local paracellular barrier without systemic electrolyte disturbances [18, 22]. This observation suggests that claudin‐3 is dispensable under physiological conditions, where the collecting duct is presumably minimally active due to relatively high dietary sodium intake. These findings align with predictions from mathematical models [39, 41] indicating that sodium reabsorption in the collecting duct contributes minimally to overall sodium handling during normal sodium intake. These models further predict that the functional importance of collecting duct increases substantially under sodium‐restricted conditions. Consistent with this, we show that claudin‐3 knockout mice exhibit a pronounced compensatory response when subjected to a low‐sodium diet. This response includes significant upregulation of α‐ and γ‐ENaC subunits and increased expression of claudin‐4, claudin‐8, and claudin‐10. One could speculate that increased ENaC, claudin‐4, and claudin‐8 abundance compensates for the paracellular sodium leakage generated by the absence of claudin‐3 along the ASDN. While forming a relatively tight barrier to sodium by decreasing Na^+^ paracellular conductance, claudin‐4 and claudin‐8 provide a less selective barrier to chloride that favors paracellular Cl^−^ reabsorption via a chloride shunt pathway, enabling Cl^−^ to follow enhanced transcellular Na^+^ reabsorption through ENaC under low‐salt conditions [24, 42, 43, 44]. Moreover, pendrin abundance remains unchanged between the two groups under low‐salt diet, suggesting that chloride transport compensation predominantly relies on the paracellular route rather than transcellular Cl^−^ exchange via pendrin.

Importantly, plasma aldosterone levels induced by LSD were similar between WT and KO mice, indicating equivalent endogenous aldosterone responses in both genotypes. In addition, increased claudin‐10 protein abundance in the TAL would increase paracellular sodium reabsorption along the lumen‐positive electrical gradient. These adaptations are not prevented by mineralocorticoid receptor blockade with spironolactone, suggesting that alternative aldosterone‐independent mechanisms contribute to maintaining sodium homeostasis in the absence of claudin‐3.

Our results expand the understanding of the role of aldosterone in renal sodium handling. Beyond its well‐established effects on ENaC‐ and Na,K‐ATPase‐mediated transcellular sodium transport [11, 12, 13, 45], aldosterone modulates the paracellular pathway through selective upregulation of claudin‐3 in addition to claudin‐8 [31]. This dual mechanism enhancing sodium reabsorption via transcellular route and preventing the back‐flux of ions to the lumen via the paracellular route appears critical to increase the efficiency of the reabsorption process. This mechanism might be crucial to maintain electrolyte balance, especially under conditions of low dietary salt intake. Future studies should be designed to elucidate the precise processes governing claudin‐3 regulation and explore whether similar mechanisms operate in human kidney epithelia, which may have significant implications for treating disorders such as hypertension and salt‐sensitive renal disease.

**FIGURE 8:**
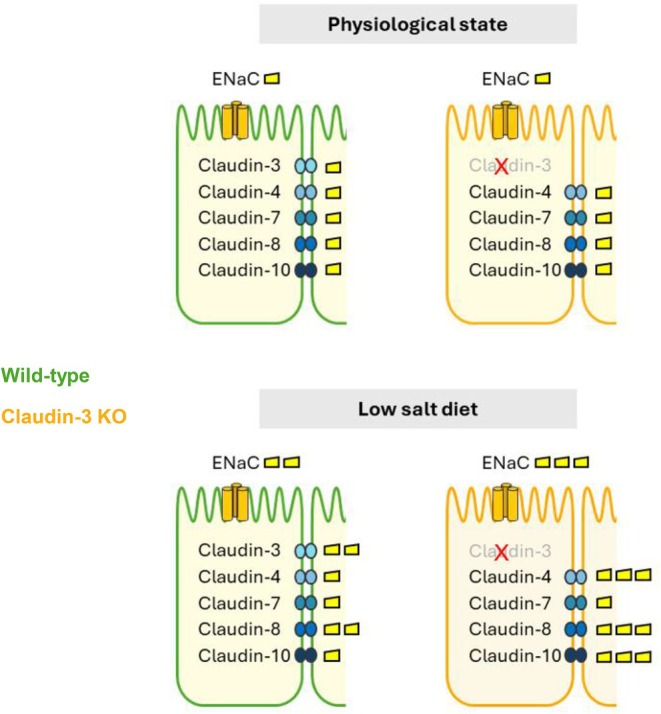
Proposed model of low‐sodium‐induced adaptations in ENaC and tight junction proteins in claudin‐3 knockout and wild‐type mice. Under physiological conditions, ENaC expression and the levels of claudin‐4, claudin‐7, claudin‐8, and claudin‐10 in the distal nephron are comparable between wild‐type (green epithelial cell) and claudin‐3 knockout (orange epithelial cell) mice. Under a low‐sodium diet, ENaC expression increases in both groups, with a significantly greater upregulation in knockout mice. Wild‐type mice respond by upregulating claudin‐3 and claudin‐8, whereas knockout mice adapt to the absence of claudin‐3 by increasing the abundance of claudin‐4, claudin‐8, and claudin‐10 as a compensatory mechanism. Despite these changes, claudin‐7 levels remain stable under both physiological and low‐sodium diet conditions in WT and KO mice. These adaptations help maintain epithelial barrier integrity and sodium homeostasis despite the absence of claudin‐3.

Collectively, these findings support the concept that modulation of paracellular permeability via tight junction proteins represents an underappreciated but physiologically significant mechanism in distal nephron adaptation to sodium depletion. A model summarizing these findings in both WT and claudin‐3 knockout mice is shown in Figure 8.

## 5 Conclusion

Our study reveals how a low‐sodium diet modulates renal sodium handling by reshaping both transcellular and paracellular pathways. We show that sodium restriction enhances claudin‐3 expression in the collecting duct, reinforcing the paracellular barrier and reducing sodium back‐leak. Although aldosterone contributes to this response, our findings indicate that the regulation of claudin‐3 and tight junction remodeling occurs as part of a broader adaptive process driven by sodium depletion. In claudin‐3‐deficient mice, compensatory upregulation of other claudins and epithelial sodium channels maintains sodium balance, illustrating the functional plasticity of the nephron. Moreover, these findings emphasize the need to consider the paracellular pathway as an active and adjustable target of hormonal and dietary regulation. Future studies should determine whether disruption of this adaptive network contributes to sodium‐wasting or salt‐sensitive hypertensive disorders, potentially unveiling new therapeutic targets for restoring renal sodium balance.

## Author Contributions

E.F. and A.S. designed the study, A.S., A.C., S.J., and A.P. carried out experiments, N.L. assisted in immunofluorescence image analysis, F.B., D.S., and M.F. provided materials and technical assistance, A.S. and A.C. analyzed the data, A.S. and A.C. made the figures, A.S. and E.F. drafted and revised the paper, all authors approved the final version of the manuscript.

## Conflicts of Interest

The authors declare no conflicts of interest.

## Supporting information


**Figure S1:** apha70216‐sup‐0001‐Figures.pdf.

## Data Availability

The data that support the findings of this study are available from the corresponding author upon reasonable request.
